# Surface texture guides egg-laying decisions in *Aedes aegypti* mosquitoes

**DOI:** 10.1242/jeb.252599

**Published:** 2026-09-21

**Authors:** Aleksandra Anoshina, Nicholas K. Tochor, Lauren Semkow, Annie Zeng, Benjamin J. Matthews

**Affiliations:** Department of Zoology, The University of British Columbia, 3051-6270 University Blvd, Vancouver, BC, Canada, V6T 1Z4

**Keywords:** Mechanosensation, Salt detection, Oviposition, Texture, Behaviour, Multisensory integration

## Abstract

Mosquitoes undergo development as aquatic larvae and pupae before emerging as terrestrial adults. Accordingly, blood-fed and mated female mosquitoes must select an appropriate egg-laying site to maximize the fitness of their offspring. Female yellow fever mosquitoes (*Aedes aegypti*) lay their eggs above the waterline of small containers or natural bodies of water, where they can remain dormant for many months until they are submerged and hatch. Here, we show that female mosquitoes use surface texture as a powerful cue to guide egg-laying decisions, selecting rougher textures over smooth when choosing among containers and when selecting specific sites within a given substrate. In addition, we identify an interaction between substrate texture and water salinity with respect to egg-laying decisions, demonstrating that female mosquitoes integrate competing cues to determine the ultimate suitability of an egg-laying site. Finally, we explore the dynamics of local egg-laying search behaviour, demonstrating that texture modulates traversal speed while mosquitoes search for appropriate egg-laying sites.

## Teaser

**Highlighted Article:** *Aedes aegypti* mosquitoes prefer rough over smooth egg-laying surfaces, revealing that tactile and environmental cues are integrated during egg-laying site selection.

## INTRODUCTION

Selecting a suitable egg-laying site is critical to fitness, particularly for mosquitoes and other insects that undergo early development in aquatic environments. The dangers associated with inappropriate site selection include a lack of food for developing larvae, the presence of predators or other natural enemies, exposure to lethal temperatures or salinities, and the disappearance of water altogether. Female yellow fever mosquitoes (*Aedes aegypti*) lay eggs in clutches of 100–150 near a variety of natural (e.g. tree holes, rock depressions) or artificial (e.g. tyres, water storage containers) bodies of water (Bentley and Day, 1989; Christophers, 2009; Day, 2016). They lay eggs above the water line, where they form a hard scleratized and melanized outer shell to prevent desiccation. Following this process, the eggs can remain viable for months to years until submerged in water (Christophers, 2009; Mayilsamy, 2019). When inundated, the eggs hatch into aquatic larvae that develop via filter feeding over the course of days to weeks. This strategy is thought to have arisen in their ancestral sub-Saharan range to prevent premature hatching in quickly dwindling water sources as the dry season approaches (Powell et al., 2018; Rose et al., 2020). As eggs can remain viable for up to 6 months, *Ae. aegypti* are a particularly pernicious and well-adapted disease vector in areas of changing climate and fluctuating precipitation (Kraemer et al., 2019, 2015).

To select a suitable egg-laying site, mosquitoes rely on a variety of long-, mid- and short-range sensory cues (Bentley and Day, 1989). These cues act on an array of sensory modalities that can operate at a distance, such as hygrosensation (Laursen et al., 2023; Bar-Zeev, 1967), olfaction and vision. Once they have identified a potential egg-laying site from a distance, female mosquitoes use contact-mediated gustatory and mechanosensory cues to generate a final decision about whether to lay an egg (Matthews et al., 2019; Afify and Galizia, 2015; O'Gower, 1957). Surface texture is a known egg-laying cue for insects broadly (Rojas et al., 2003; Renwick and Chew, 1994), including for aedine mosquitoes (Beckel, 1955; Wallis, 1954; Momen et al., 2025; Chadee et al., 1995; Fay and Perry, 1965; Wong et al., 2011). For example, Fay and Perry (1965) found that *Ae. aegypti* females show a significantly stronger preference for brown blotting paper and brown bag paper over white filter paper or aluminium foil. In another field-based study, Chadee et al. (1995) demonstrated that female *Ae. aegypti* prefer to lay eggs on rough substrates, which they suggest may facilitate resting and egg laying. Finally, O'Gower (1957, 1963) found that *Ae. aegypti* use a variety of chemical, visual and tactile cues to guide egg laying, with tactile cues often dominating over other modalities when standing water is available. However, the mechanism by which tactile cues guide egg-laying decision-making processes is not well understood.

To better understand the role of mechanosensory cues in *Ae. aegypti* egg-laying behaviour, we first sought to characterize the impact of surface texture (roughness) in isolation from other egg-laying cues in controlled laboratory assays. We hypothesized that altered substrate texture would impact egg-laying behaviour and preference, predicting that rougher textures would be preferred over smooth, and that female mosquitoes would employ search strategies that maximized the likelihood that they would lay eggs on rough surfaces. To test this, we performed a series of two-choice preference assays at different spatial scales to determine how different textures influenced egg-laying preference with respect to site selection both between containers and within individual containers. We explored differences in how eggs were deposited relative to one another depending on whether eggs were deposited on rough or smooth surfaces and investigated the interaction between rough texture (a positive cue) and elevated salinity (a negative cue). Finally, we performed video analysis to determine how surface roughness influences mosquito locomotion during egg-laying bouts.

## MATERIALS AND METHODS

### Experimental model and organisms

*Aedes aegypti* (Linnaeus in Hasselquist 1762) mosquitoes (Orlando strain; Matthews lab stock) were reared and maintained in an insectary at 25–28°C, 70–80% relative humidity with a 14 h:10 h light:dark cycle. Mosquito eggs were hatched under vacuum for 30 min and reared in dechlorinated Vancouver tap water at densities of 250 larvae per 2–2.5 l of water and temperature of 27°C while being fed ground fish flakes (TetraMin Tropical Flakes, Tetra) as needed.

Pupae were transferred to 30 cm^3^ Bugdorm cages at densities of 300–350 animals per cage. Adults were provided with 10% sucrose *ad libitum* for 9–13 days before being blood fed with defibrinated sheep's blood (Hardy Diagnostics) in Parafilm-covered glass feeders (Chemglass, 50 mm diameter) with 37°C water circulating through an outer water jacket or a Hemotek blood-feeding apparatus to maintain temperature. After blood feeding, engorged females were visually selected and transferred to a new cage with sucrose and used for behaviour experiments 4 days after the blood meal. Females were not used beyond their first gonotrophic (egg-laying) cycle.

### Preparation of textured agarose plates

A 3% w/v molten agarose solution (BioBasic, Agarose A) was made by dissolving 3 g powder per 100 ml of dechlorinated water and microwaved until all agarose was dissolved and the solution was bubbling; this was cooled at room temperature until warm to the touch (∼60°C). Large Petri dishes (150 mm diameter, Fisher Scientific) were lined with sandpaper disks of the desired grit (3M Pro Grade No-Slip Sandpaper, ranging from 30.2 μm to 190 μm average particle size; see Table 1), glued textured-side up to the dishes; 75 ml of warm 3% agarose solution was poured into the dishes and allowed to set until firm. Circles of clear, solidified agarose were cut from these dishes with a sterile blade and transferred to smaller 60 mm Petri dishes (Fisher Scientific) with the textured side up. The same procedure was repeated for smooth plates but using the reverse (smooth) side of a sandpaper disk to imprint a flat surface. For split-dish assays, a 150 mm Petri dish was used as a mould, with two semicircles of sandpaper glued to the bottom to transfer texture to each half of the assay.

**Table 1.** Conversion between commercially available ‘grit’ of sandpaper and average particle size used in the study

Sandpaper grade	Average particle size (μm)
Smooth	0.0
P500	30.2
P400	35.0
P360	40.5
P320	46.2
P220	68.0
P180	82.0
P150	92.0
P120	115.0
P80	190.0

Values of average particle size are derived from the manufacturer's data for the brand of sandpaper used (3M Pro Grade No-Slip Sandpaper).

### Two-choice egg-laying assay

White restaurant supply buckets (85 oz/∼2.5 l volume) with a fine mesh top covering were used as arenas for egg-laying assays. Two Petri dishes (60 mm in diameter) were mounted on angled support structures (tilted at ∼20 deg) facing towards the centre of the arena, 1–2 cm apart, and 2 ml of dechlorinated tap water was pooled at the bottom of each dish. In trials where elevated salinity was used as the liquid in a given dish, NaCl (Fisher Scientific) was dissolved in dechlorinated tap water to a predicted osmolality of 100 mOsm kg^−1^ or 200 mOsm kg^−1^, verified by an osmometer. Ten gravid females were added to each arena, which was then placed in the insectary for 22–24 h at 70–80% humidity and 25–28°C. Placement of texture treatments was randomized within each arena and the arenas were distributed randomly in the insectary.

### Photographing of egg papers

Eggs were retrieved on Petri dishes after 22–24 h of egg laying and counted under a dissecting scope by hand using a spatula to move the eggs as they were counted. Raw egg counts were recorded for each pair of dishes. For automated counting algorithms, dishes were photographed using an SLR camera (Nikon D7000) with a macro lens (Tamron 90 mm F/2.8 Di SP MACRO 1:1) at a set height of 44 cm. Dishes were illuminated using an LED backlight to enhance the contrast of the melanized eggs against the agarose. Images were cropped using Adobe Photoshop CS6 to form 3200×3200 pixel-sized circles of the inner portion of the dish along the edges of the agarose substrate.

### Data analysis

Eggs were manually counted and a preference index (PI) was calculated for each two-choice assay using the following formula:

(1) $$\mathrm{PI}=\frac{\mathrm{eggs}\;\mathrm{on}\;\mathrm{side}\;1\;-\mathrm{eggs}\;\mathrm{on}\;\mathrm{side}\;2}{\mathrm{eggs}\;\mathrm{on}\;\mathrm{side}\;1\;+\mathrm{eggs}\;\mathrm{on}\;\mathrm{side}\;2}.$$

For split-dish two-choice assays, eggs within 3 mm of the midline (where the two textures met) were not considered in calculating the preference index. The data were visualized with R (https://www.r-project.org/) and RStudio (https://posit.co/) using the following packages: ggplot2 (Wickham, 2016), drc (Ritz et al., 2015), and tidyverse (Wickham et al., 2019). Details of specific visual representations and statistical tests used are reported in the appropriate figure legends.

For clumping analysis, a separate two-choice assay experiment was repeated using the setup described above. Representative, high-resolution images of dishes of tested textures were photographed with an SLR camera (Nikon D7000) with a macro lens (Tamron 90 mm F/2.8 Di SP MACRO 1:1) at a set height of 44 cm and analysed for egg clustering using FIJI/ImageJ (Schindelin et al., 2012). A circular image was turned to an 8-bit image. Egg centroids were first selected with Process>Find Maxima… with ‘Prominence’ set above 100.00, the ‘Light background’ box checked, and ‘Point selection’ as the output type. Then, the multi-point selection tool was used to go over the image manually to select any non-selected centroids and delete erroneous selections of non-egg particles. Once all centroids were selected, a macro (https://imagejdocu.tudor.lu/macro/multiple_points) was used to export the points into .csv and Excel file formats. A complementary macro from the same source was used to import the points back onto the image.

To determine the average distance to the six closest neighbours of each centroid, a plugin was used as described in Haeri and Haeri (2015). With points selected on the image, Analyze>Measure was used followed by the ND plugin with ‘6’ as the number of neighbours.

### Imaging and analysis of temporal dynamics

For live imaging experiments, mosquitoes were reared as above and blood-fed 96 h prior to each assay. Fully engorged mosquitoes were selected and provided with sucrose *ad libitum* prior to recording. Imaging sessions took place during the mosquito's subjective day (Zeitgeber time, ZT +6, +9 or +12 h of a 14 h:10 h light:dark cycle).

Mosquitoes were provided with 3% agarose, prepared as described above for split-dish assays (190 μm average particle size), from which a 65 mm^2^ rectangular slab was carved with a custom 3D printed cutter, with equally sized smooth and rough halves. These agarose slabs were stored in dechlorinated water until use.

For each imaging session, a single mosquito was cold-anaesthetized and then placed into a small custom-made acrylic arena (approximately 65×65×50 mm) with the agarose slab at its centre, tilted ∼20 deg; 2 ml of dechlorinated tap water was added to the bottom of this slanted arena to stimulate egg laying. The lid to a 90 mm Petri dish was placed over the arena to contain the mosquito, and the entire apparatus was placed within a blackout box (no visible light); the arena was illuminated with infrared LEDs (940 nm). A 120 min recording was triggered using the GPIO pins on a Basler a2A2448-75umPRO camera with a Marshall Electronics 11–50 mm f1.4 cs-mount camera lens (CS1150-8MP) with a 3D-printed aperture insert with a constant 3 mm diameter.

Centroid tracking of mosquitoes in each video was accomplished using SLEAP v1.4.2 (Pereira et al., 2022) and egg-laying events were manually scored. A model skeleton of the mosquito was produced in an iterative process alternating between manual training and automated model refinement. All point data were calculated using the centroid of the mosquito's thorax, which was considered the centroid of the mosquito for tracking purposes. These centroid coordinates were exported as a csv and processed using R v4.4.1 (https://www.r-project.org/) for further analysis. The position of the mosquito and each egg was calculated relative to the midline, and a rolling average of velocity was calculated with a window of 0.5 s.

## RESULTS

### Rough substrate texture is a positive egg-laying cue

To test texture preference during egg laying, we created a two-choice egg-laying assay where 10 blood-fed and mated female mosquitoes were placed in a small chamber with access to two 60 mm Petri dishes, each with a small pool of water and an agarose egg-laying substrate generated by letting molten agarose harden on top of sandpaper of varying roughness (Fig. 1A). *Aedes aegypti* showed an almost universal preference for all textured substrates over a smooth alternative. The strength of this preference was correlated with the degree of roughness of the substrate, i.e. it increased with the average particle size of the substrate surface (Fig. 1B; *n*=9–26 assays per condition). The relationship between egg-laying preference and surface texture can be described with a log–logistic function (Fig. 1C and see Materials and Methods). Analysis of egg numbers revealed a weakly negative relationship between the roughness of the textured option and total egg number across both plates of the assay (Fig. 1D).

**Fig. 1.**
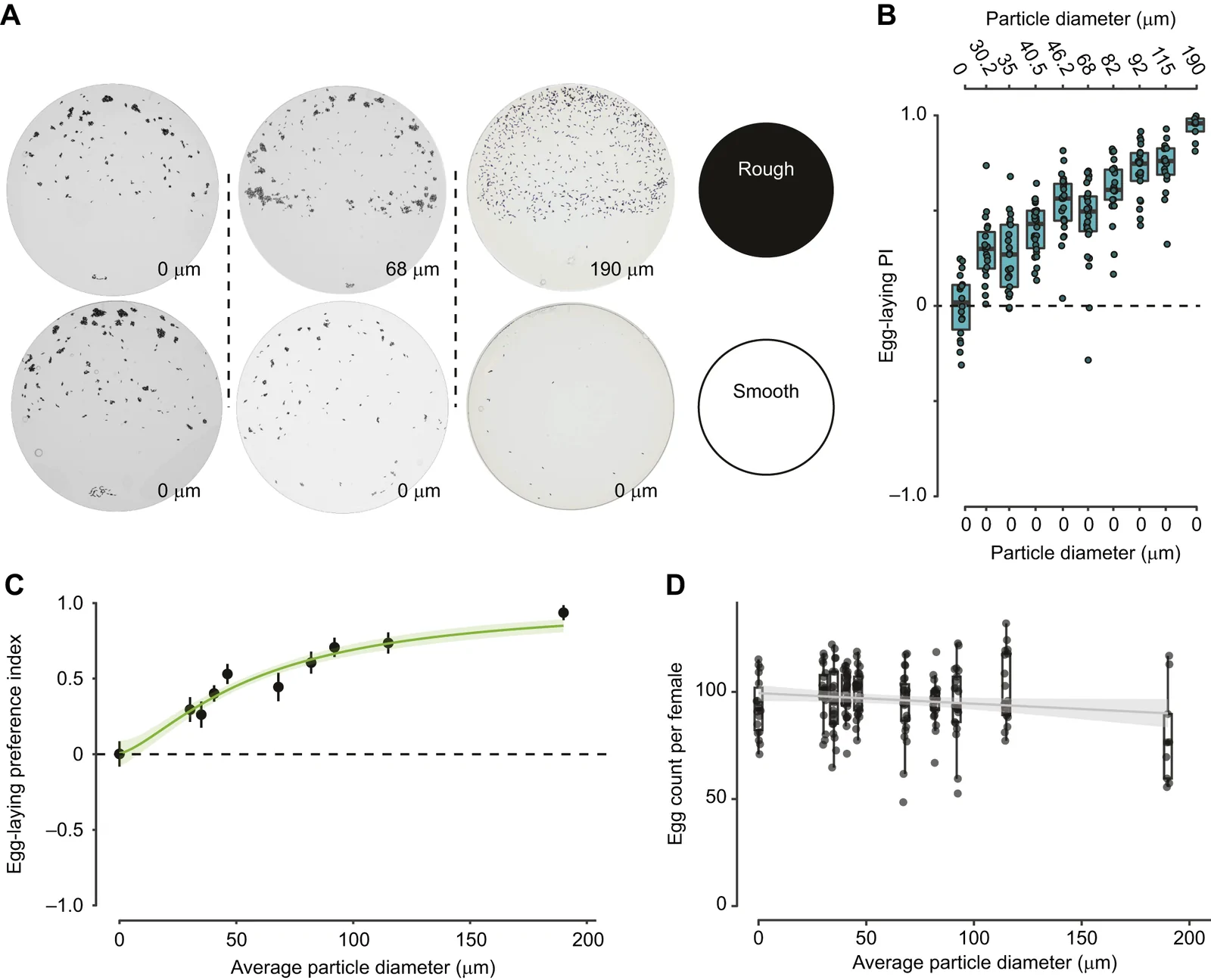
**Mosquitoes prefer rough egg-laying substrates.** (A) Representative images of *Aedes aegypti* eggs deposited on 60 mm agarose plates imprinted with sandpaper of different average particle diameter (two-choice assay with the textures indicated, left to right: 0 μm versus 0 μm, 68 μm versus 0 μm, and 190 μm versus 0 μm). (B) Egg-laying preference index (PI) measured in two-choice assays in which females chose between a smooth agarose substrate and an agarose substrate textured with sandpaper of increasing average particle diameter. A positive PI indicates preference for the textured substrate (top *x*-axis) over the smooth substrate (bottom *x*-axis), and each assay consisted of 10 blood-fed mosquitoes. Box plots show median, upper and lower quartiles and 1.5× the interquartile range; circles are individual data points. A one-way analysis of variance (ANOVA) followed by Tukey honestly significant difference (HSD) pairwise tests indicated that all textured surfaces show increased preference relative to control. (C) Four-parameter log–logistic function (fixed upper bound of 1 and lower bound of 0) fitted to the egg-laying preference data shown in B [slope=−1.4394; median effective dose (ED50)=57.20 μm]. The green line represents the curve fit, while light green shading represents the 95% confidence interval of the fit. Circles and whiskers are means and 95% confidence interval of the original data. (D) Total number of eggs laid per female in the two-choice assays (smooth versus textured plates with the average particle size indicated on the *x*-axis) fitted with a linear regression (slope=−0.04960, adjusted *R*^2^=0.01453, *P*=0.047). *n*=9–26 assays per condition.

### Texture guides egg-laying decisions at the small scale

To determine the spatial scale at which texture preference acts, we created a variant of our two-choice assay, in which semi-circles of textured agarose were placed directly adjacent to one another in a ‘split-dish’ format (Fig. 2A). When there was a different roughness on each side, we observed a preference for the half of the dish that contained the rougher texture (Fig. 2B; *n*=5–10 mosquitoes per pairing, 80 in total), which tracked the relative difference in average particle diameter (Fig. 2C). Interestingly, the model fitted to the relative texture data is qualitatively similar to the model fitted to the two-choice assays (Fig. 1C), suggesting that egg-laying *Ae. aegypti* mosquitoes maintain a straightforward preference for a relatively rougher surface while choosing a substrate both within and between containers.

**Fig. 2.**
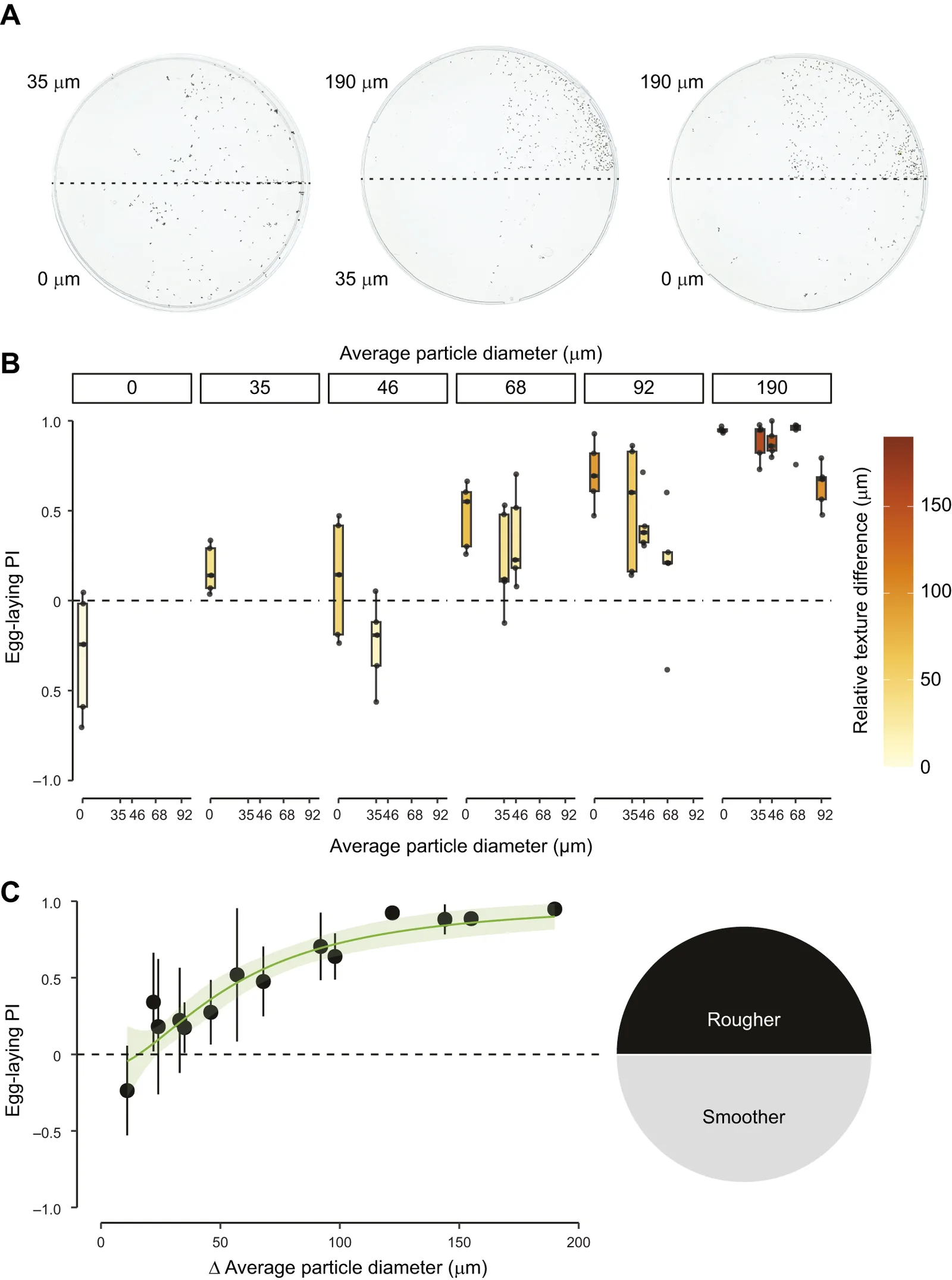
**Rough texture preference is maintained at small scales.** (A) Representative images of split Petri dishes used to assay egg-laying preference between substrates differing in average particle size. Each dish contained two halves, each patterned with a texture with a defined average particle diameter as indicated. (B) Egg-laying PI of mosquitoes offered split-Petri dishes with the indicated textures. For each boxplot, the relative texture difference of the two halves is represented by the colour scale on the right. (C) Log–logistic function (fixed upper bound of 1 and lower bound of 0) fitted to the egg-laying preference data shown in B [slope=−2.169; ED50=62.77 μm]. The green line represents the curve fit, while light green shading represents the 95% confidence interval of the fit. Circles and whiskers are the means and 95% confidence interval of the original data. Note the similarities to the function from Fig. 1C. *n*=5–10 per condition.

### Eggs are ‘clumped’ on smooth surfaces

In instances where mosquitoes laid their eggs on smooth surfaces, they appeared to ‘clump’ their eggs together as opposed to evenly spreading them across the surface (Fig. 3A). To quantify the degree of egg clustering, we calculated the distance of each eggs' closest neighbours (*n*=2109–4402 eggs per texture). Eggs deposited on smoother surfaces were more tightly clustered, whereas eggs laid on surfaces with rougher textures were more likely to be distributed singly (Fig. 3B). The tendency to lay eggs in ‘clumps’ on smoother textures was demonstrated by analysing the mean distance between an egg and its six nearest neighbours (Fig. 3C). We note that the data are heavily skewed, with peak average distances at the short end and few points at the long end, representing single eggs scattered around the larger egg clusters.

**Fig. 3.**
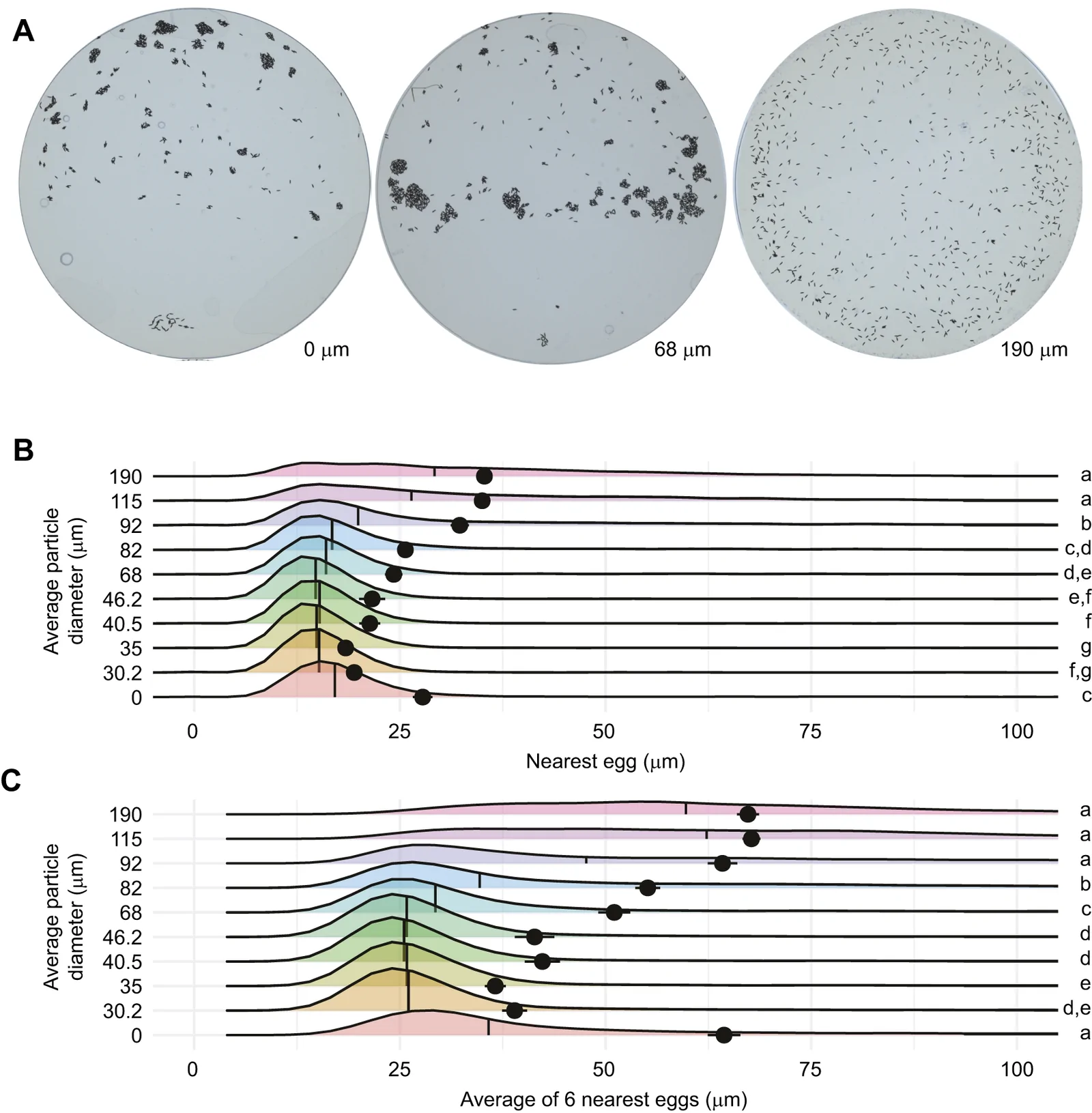
**Eggs are laid in clumps on smooth surfaces.** (A) Representative plates with the indicated texture (average particle diameter) reveal differential levels of ‘clumping’ across textures. (B,C) Quantification of the spatial arrangement of eggs relative to (B) their single nearest neighbour and (C) the average of the six nearest neighbours. For B and C, the vertical line indicates the median of the distribution, while the circle and whiskers represent the mean and 95% confidence interval. Letters indicate significant differences (*P*<0.05) between group means as determined by one-way ANOVA followed by Tukey honestly significant difference (HSD) pairwise tests. *n*=2109–4402 eggs analysed per condition.

### Interaction of substrate texture and water salinity

We next wondered whether the presence of an aversive egg-laying cue (elevated salt) would influence texture preference in *Ae. aegypti* mosquitoes. We first repeated our split-dish assays but instead of providing fresh water, we used a solution of 200 mOsm kg^−1^ NaCl, which is an aversive cue (Matthews et al., 2019). We found that the preference for rougher textures remained in the presence of salt (Fig. 4A; *n*=5 per condition) and in fact was nearly identical to the curve generated in fresh water (Fig. 4B), suggesting that mosquitoes can evaluate the relative value of multimodal cues independently when determining ideal egg-laying sites. To test the interaction between salt (a negative egg-laying cue) and rough texture (a positive egg-laying cue), we set up two-choice assays between smooth and salt-free substrates and rough and salty substrates at two different concentrations. We found a significant effect of salt concentration on egg-laying preference (Fig. 4C; *n*=5 per condition), with the higher concentration of salt completely abolishing the positive preference for rough textures. The degree to which salt reduced the preference for rough textures did not differ with roughness (Fig. 4D). This indicates that mosquitoes can integrate two competing cues, but that at higher concentrations of salt, texture is not relevant.

**Fig. 4.**
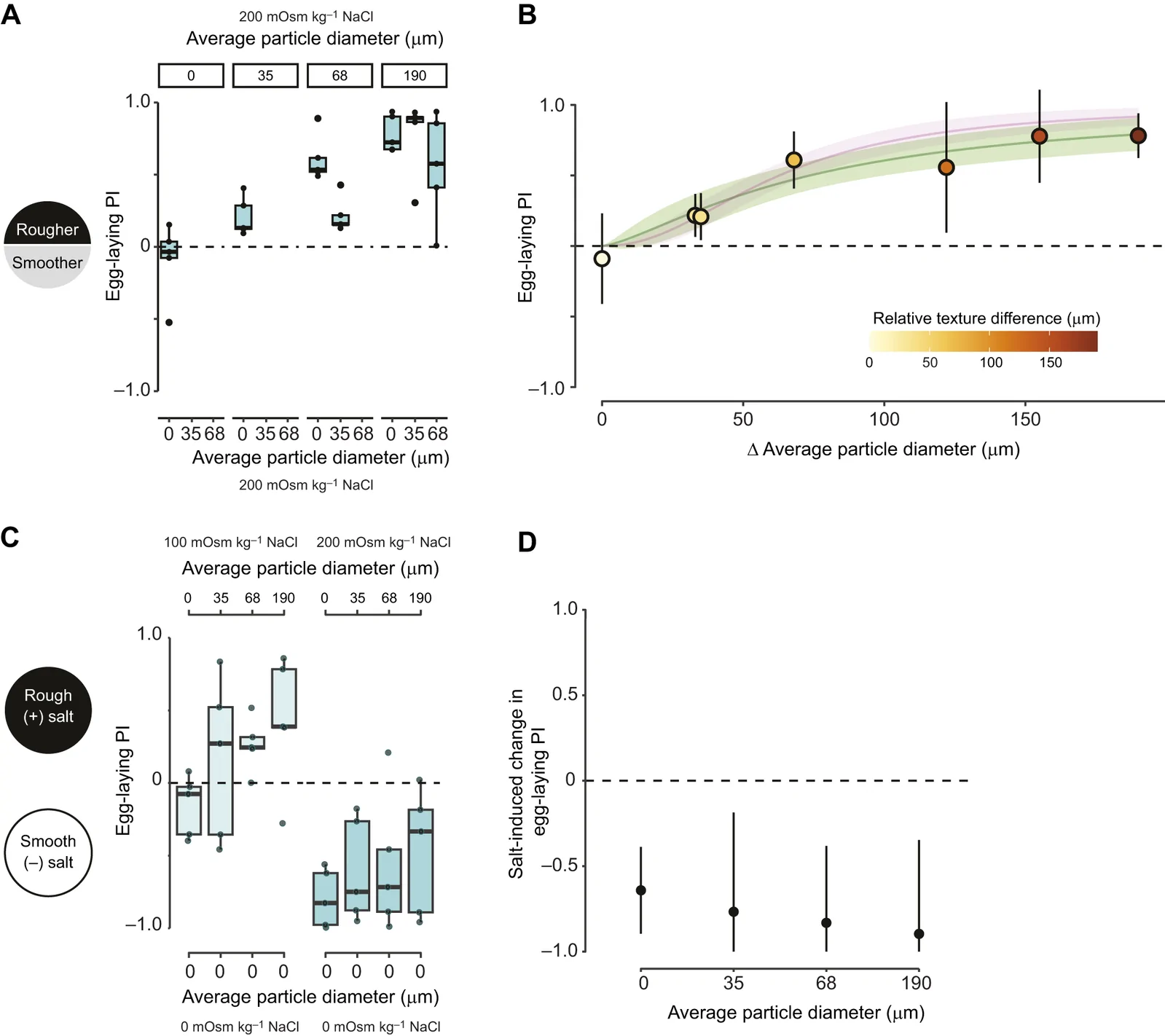
**Texture and salt are interacting egg-laying cues.** (A) Split-dish texture assays performed with 200 mOsm kg^−1^ NaCl solution for both choices. (B) Log–logistic function (fixed upper bound of 1 and lower bound of 0) fitted to the egg-laying preference data shown in A [slope=−1.407; ED50=73.63] shown in green; log–logistic function fit from experiments with no salt in Fig. 2B are overlaid in magenta. (C) Two-choice egg-laying assays comparing the preference for smooth surfaces with fresh water and rough surfaces with salt water at 100 mOsm kg^−1^ (left) or 200 mOsm kg^−1^ (right). A two-way ANOVA with the contrast of (PI∼salt×texture) revealed a significant effect of salt (*P*=2.9×10^−7^). (D) The difference (mean and 95% confidence intervals) in PI in 200 versus 100 mOsm kg^−1^ NaCl for the textures shown. *n*=5 per condition in A and C.

### Surface texture changes locomotor behaviour during egg laying

We next asked whether surface texture plays a role in movement dynamics during mosquito egg laying by filming individual mosquitoes on a slab of agarose in which one half was smooth and the other was textured with 190 μm sandpaper (Fig. 5A). We tracked the mosquito's position and manually scored egg-laying events for nine mosquitoes over 120 min and found that mosquitoes routinely traversed the boundary between smooth and textured sides of the plate, a sign of active exploration and local search during egg laying (Fig. 5B,C).

**Fig. 5.**
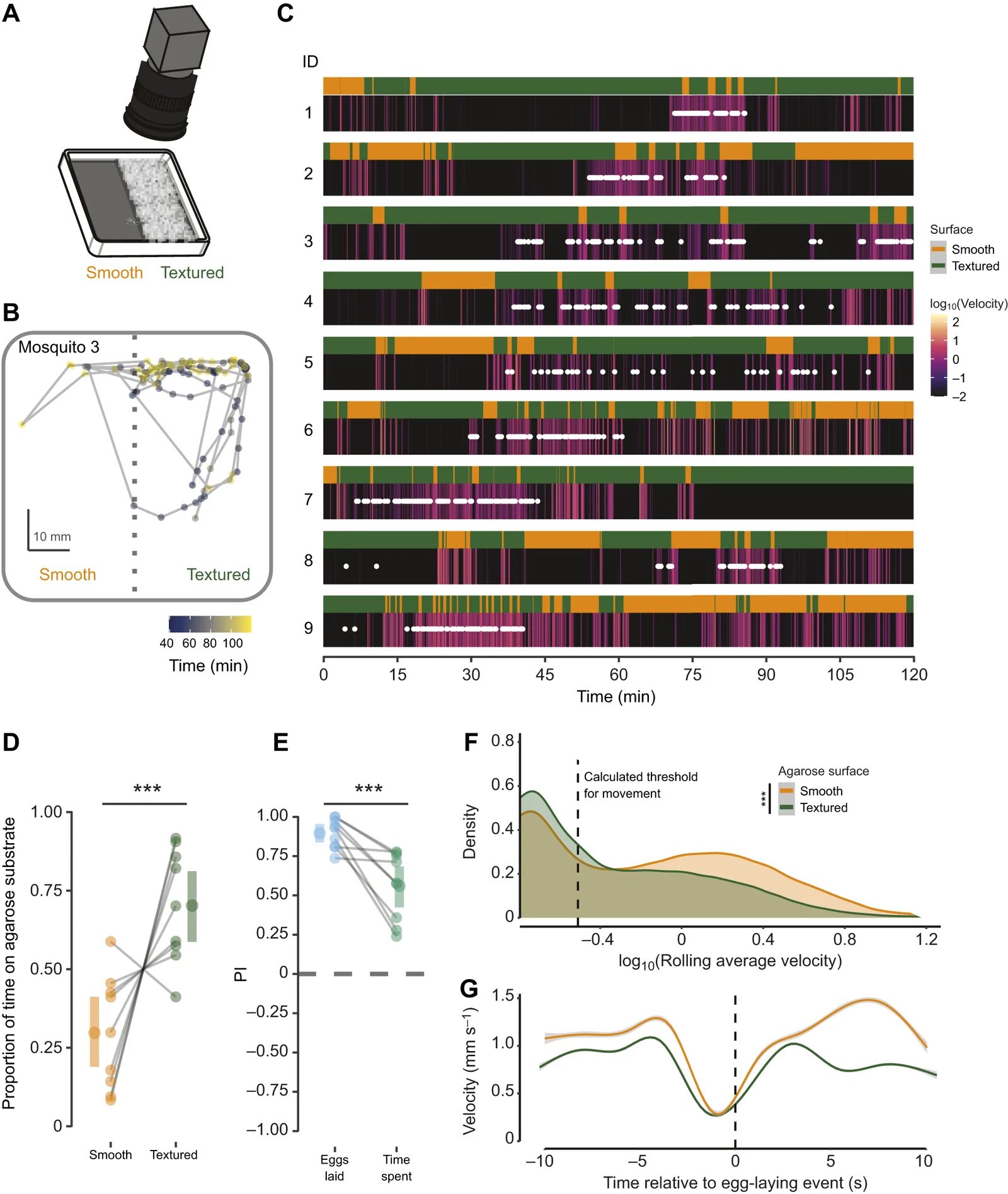
**Substrate texture shapes mosquito movement during egg laying.** (A) Schematic diagram of the behavioural arena and tracking setup used to record individual gravid female mosquitoes during a 120 min egg-laying session (96 h post-blood meal). Females were allowed to move freely between smooth and textured agarose substrates. (B) Example egg-laying events from a representative individual (ID=3) overlaid on the arena, with positions of eggs (circles) coloured by instantaneous velocity. (C) Raster plot summarizing behavioural state and position across time for individual mosquitoes (ID, identification number for an individual mosquito) during the recording session. Coloured segments indicate substrate occupancy (smooth/textured; top) and movement speed (velocity, mm s^−1^; bottom) over time. White circles indicate egg-laying events. (D) Proportion of time spent on smooth versus textured substrates for individual mosquitoes, shown as paired measurements for each individual mosquito. (E) Relationship between time spent on each substrate and the number of eggs laid, shown for individual animals. (F) Distribution of rolling-average (window=0.5 s) velocity (mm s^−1^) on smooth and textured substrates, plotted on a log_10_ scale. The vertical dashed line represents the threshold used for calculating ‘movement’ based on the average velocity of representative clips in which mosquitoes were scored as ‘stationary’. (G) Rolling-average velocity profiles (window=0.5 s; mean and 95% confidence intervals) aligned to egg-laying events across all mosquitoes, shown separately for smooth and textured agarose. *n*=9 recordings and 658 eggs laid. ANOVA followed by Tukey honestly significant difference *post hoc* tests were performed for D and E; ***P<0.001. Kruskal–Wallis test for *F* indicates a significant relationship between velocity and substrate texture across the entire dataset (*P*<0.001).

We found that mosquitoes spent significantly more time on the textured half of the plate (Fig. 5D) but, when expressed as a preference index, their place preference was significantly lower than their egg-laying preference (Fig. 5E). This indicates that egg-laying preference reflects an increased probability of laying eggs on rough textures and is not simply a product of preferring to spend time on rough surfaces. When examining a mosquito's velocity on each half of the plate, we found that they moved significantly faster on smooth agarose (Fig. 5F). When examining movement dynamics locked to egg-laying events, we found that mosquitoes showed a characteristic ‘pause’ ∼1 s prior to the appearance of an egg, followed by a rebound in velocity that was significantly higher when animals were on smooth as compared with textured substrate (Fig. 5G). Together, this suggests that mosquitoes behave differently on different textured surfaces.

## DISCUSSION

### The importance of mechanical cues in egg-laying decisions

Our results highlight the role of interacting cues in dictating egg-laying site selection. While some cues guide mosquitoes to bodies of water from a distance, contact-mediated evaluation of water and substrate quality represents a final ‘go/no-go’ decision checkpoint prior to egg deposition. In this framework, evaluation of water quality (e.g. for appropriate salinity and absence of predators) unlocks a final search behaviour which culminates in the identification of a particular patch of surface on which to affix each egg.

By choosing textured surfaces, the female mosquito may be maximizing surface area of contact between egg and substrate, which could reduce the likelihood of an egg becoming prematurely dislodged before submergence. Future experiments to determine the force required to dislodge an egg on a smooth versus a textured surface could directly demonstrate this relationship. In addition, depositing eggs in grooves and other features may buffer them from changes in temperature or humidity (Russo, 1978) or provide protection from predators (James, 1966; Byttebier and Fischer, 2019). Overall, ensuring adherence to the substrate and protection from the elements could be critically important for the ability of *Ae. aegypti* mosquitoes to repopulate areas of their range which experience strong seasonality in precipitation, temperature or other climatic factors.

In other species, the mechanical properties of a particular substrate can influence egg-laying decisions, reflecting the complex relationship between an egg, the substrate and other considerations of egg laying. For example, the spotted wing drosophila, *Drosophila suzukii*, which lays eggs inside ripening fruit, does so in a curvature-dependent fashion (Akutsu and Matsuo, 2022), which might reflect the geometry of their ideal host fruits*.* Across European damselflies, which lay their eggs inside plants, there is a correlation of the puncture forces required to penetrate a preferred plant host and the stiffness of the oviopositor (Matushkina and Gorb, 2007). In the oriental armyworm, *Mythimna separata*, adult females select ‘slits’ in a substrate to lay their eggs in, a strategy which may help them defend their eggs from parasitoid wasps (Ma et al., 2025). Based on our results, we predict that any insect that would benefit from the adhesion of an egg to a substrate for prolonged periods of time, and particularly that show delayed hatching such as mosquito eggs awaiting the next rainfall, might utilize texture as a positive egg-laying cue similarly to *Ae. aegypti*. In this framework, surface area contact between the egg and the substrate would work in conjunction with ‘egg glues’ and other mechanisms to promote the adhesion of egg to substrate (Hinton, 2013).

We found that mechanical (texture) and chemical cues (salinity) interact in a hierarchical fashion to guide overall egg-laying decisions in *Ae. aegypti*. This is true in other insects as well, including *D. suzukii*, where the effect of surface hardness can supersede a preference against chemical cues derived from microbes (Sato et al., 2021). Future studies on egg-laying preference should thus consider the potential for combinatorial or hierarchical interactions between egg-laying cues, positive and negative.

### The cellular and molecular basis of texture discrimination during egg laying

As the mosquito pauses just before laying an egg, we posit that a close evaluation of surface texture (likely in conjunction with other cues including surface moisture and hardness) guides the selection of a specific location for each egg. This final search likely involves the deflection of mechanosensory hairs on the ovipositor of the mosquito (Rossignol and McIver, 1977), which would allow them to identify appropriately sized divots or other features in which to deposit each egg. In *Drosophila*, labellar and tarsal mechanoreceptors also appear to be involved in texture and stiffness discrimination during egg laying (Zhang et al., 2020). This raises the possibility that mosquitoes use sensory appendages beyond the ovipositor to evaluate surface texture.

Mechanosensation in insects relies on receptors from several gene families including those encoding piezo, ppk, Tmc and TRP channels (Hehlert et al., 2021). The mosquito ovipositor and tarsi express members of each of these gene families (Matthews et al., 2018, 2016; Goldman et al., 2025), which in other species, have been implicated in texture-driven egg-laying behaviour (Zhang et al., 2020; Wu et al., 2019; Guo et al., 2020; Zhang et al., 2025). Identification of mechanosensory receptors and cell types responsible for egg-laying site selection is an important goal of future research.

### Relevance to disease transmission

Egg-laying decisions can have widespread and important impacts on mosquito-borne disease transmission. The yellow fever mosquito, *Ae. aegypti*, is an important vector of arboviral pathogens that cause dengue fever, chikungunya, Zika, yellow fever and other diseases. Combined, these diseases kill tens of thousands of people every year. Pathogen acquisition by vector mosquitoes requires multiple blood meals, and the link between egg laying and the release of blood meal-induced host-seeking suppression in some mosquitoes, including *Ae. aegypti*, makes egg laying an important component of arboviral disease spread (Duvall, 2019). Furthermore, the search for egg-laying sites appears to drive the dispersal of mosquitoes post-blood meal (Reiter et al., 1995). Additionally, we propose that one role of a preference for rough surfaces is to maximize the surface area contact between egg and substrate, allowing eggs to persist above the water line, avoiding predation and premature hatching and ultimately increasing population density of future generations. We propose that identifying cues and describing the mechanisms guiding egg-laying decisions can help build better tools for mosquito surveillance and control, including gravid ovitraps (Momen et al., 2025; Perera et al., 2025).

### Comparative considerations and limitations of the present study

In the present study, we chose to use relatively small (60–100 mm diameter) surfaces made from 3% hydrated agarose gel as a proxy for egg-laying sites to investigate the preference of a common laboratory strain of *Ae. aegypti*. This choice of substrate allowed us to carefully control additional non-texture parameters of the egg-laying site (e.g. visual cues, size of container, substrate water content and olfactory/taste cues). We chose to utilize straightforward two-choice assays in which mosquitoes were presented with either two distinct containers with single textures (e.g. smooth versus 190 μm) or split-dish assays with a single surface divided into two discrete zones (e.g. between 68 and 190 μm), building up a comprehensive dataset of relative textural differences (*n*=80 in total; 5–10 per pairing). Building on these ‘simple’ choices, future work examining more complex surfaces (e.g. surfaces with a gradient of textures or those with distinctive features, such as grooves or edges) will be important as we work towards understanding the full landscape of mechanosensory-guided egg laying in *Ae. aegypti*.

There are over 3600 species of mosquito, many of which exhibit unique egg-laying strategies. While the focus of our present study, *Ae. aegypti*, lays desiccation-resistant eggs singly above the water line in small containers, other mosquito species lay eggs directly on water in rafts, deposit eggs deep into moist soil substrates or even deposit eggs aerially into bodies of water (e.g. *Sabethes* mosquitoes) (Bentley and Day, 1989; Day, 2016; Vieira et al., 2020 preprint). In addition to between-species differences, there are often notable differences in egg-laying strategy and preference within strains or subspecies of mosquito. For example, the ancestral form of *Ae. aegypti*, *Ae. aegypti formosus*, can be found in rural and forested regions of Africa, where it can utilize natural breeding sites including tree holes and rock pools as well as artificial containers (Xia et al., 2021). As *Ae. aegypti* became increasingly associated with human habitation, their dependence on artificial containers such as tyres, buckets or water storage containers may have increased their ability to breed year-round (Rose et al., 2020; Xia et al., 2021). We note that many of these artificial containers may be constructed of smooth material (e.g. glass or plastic), which, based on the results of the present study, we would predict to be sub-optimal (at least from a textural standpoint). Thus, one intriguing possibility is that the willingness of certain strains of *Ae. aegypti* to ‘tolerate’ these sub-optimal textures and thus the behavioural flexibility to utilize novel oviposition sites may have contributed to their ability to expand into urban environments and thus become more invasive. Finally, we note that the natural breeding sites of *Ae. aegypti* will contain surfaces of differing size that additionally vary in hardness, water content and other surface material parameters in addition to texture. A full description of the sensory ecology of egg laying in a particular species or strain of mosquito will require a thorough examination of the relative importance and precise role, if any, of surface texture and mechanosensation in conjunction with other relevant cues.
